# Adherence to Insulin Treatment in People With Type 1 or Type 2 Diabetes in Primary Health Care in a Brazilian City: A Cross‐Sectional Study

**DOI:** 10.1002/hsr2.72105

**Published:** 2026-03-13

**Authors:** Lívia Maria Ferrante Vizzotto Consoli, Rosiane Chiaroti, Lorrana Luysse dos Anjos Assis, Vanessa Patrícia Pereira Motozo, Francisco Barbosa‐Junior, Rinaldo Eduardo Machado de Oliveira

**Affiliations:** ^1^ University of São Paulo, Ribeirão Preto Medical School, Postgraduate Program in Public Health Ribeirão Preto São Paulo Brazil; ^2^ University of Brasilia Faculty of Health Sciences and Technologies, Postgraduate Program in Pharmaceutical Services and Policies Brasilia Federal District Brazil

**Keywords:** diabetes mellitus, epidemiology, insulin, medication adherence, public health

## Abstract

**Background and Aim:**

Non‐adherence to diabetes medication treatment is a recognized public health problem that can lead to unfavorable clinical, humanistic, and economic outcomes. This study aimed to analyze adherence to insulin treatment in people with type 1 diabetes or type 2 diabetes in primary health care in a Brazilian municipality.

**Methods:**

This cross‐sectional study was developed with 152 participants and a simple probability sample. We collected blood and urine samples, performed ophthalmological fundus examinations using a portable retinal camera on a smartphone, and conducted face‐to‐face interviews. Adherence was measured using the Insulin Treatment Adherence Measure, a version validated in Brazil.

**Results:**

A higher proportion of women (63.2%), without private health plan (84.2%), with a medically diagnosed type 2 diabetes (93.3%), inadequate glycemic control (76.9%), and without diabetic retinopathy (71.7%) was observed. The prevalence of insulin therapy adherence was estimated at 47.4% (95% CI 39.4–55.3). Higher insulin therapy adherence was observed among males, self‐reported Nonwhite race/ethnicity, and those diagnosed with systemic arterial hypertension. A negative association was observed with alcohol abuse, amputation, and among those with private health plan (*p* < 0.05).

**Conclusion:**

Adherence to insulin treatment was low, and this scenario can lead to complications with various impacts, including increased healthcare costs. The associated variables evidenced the need for drug therapy management, incorporating multidimensional care for people with diabetes, to enable disease control in primary health care.

## 1 Introduction

Diabetes is a chronic, noncommunicable condition with high prevalence worldwide, and a lack of control can lead to irreversible harm [1]. The increasing rate of population aging and the reduced number of people adopting a healthy lifestyle can contribute to the onset of the disease [2]. In Brazil, the scenario is worrying. The surveillance study of risk factors and protection against chronic diseases by telephone survey (Vigitel) showed that the frequency of adults who self‐reported a diagnosis of the disease increased from 5.5% in 2006 to 12.9% in 2024 [3].

Diabetes treatment, based on regular medication use, a balanced diet, regular exercise, and health education, is crucial for glycemic control, complication prevention, and improving and maintaining quality of life [4]. Difficulties in treatment adherence are believed to be related to the drug therapy complexity, access, and low adherence. This backdrop is well‐known in the daily lives of healthcare teams and is a strong predictor of undesirable clinical outcomes [5].

Literature provides us with diverse concepts of treatment adherence, and there is no consensus. In 2003, the World Health Organization (WHO) presented a classic multidimensional model of adherence, with five integrated dimensions that influence this process, namely, socioeconomic characteristics, disease, therapy, health services, and personal aspects. The WHO estimated the prevalence of adherence to chronic treatment in the general population at 50% [6].

Non‐adherence to insulin therapy is a recognized public health problem [6, 7]. Some studies have shown that adherence to treatment with oral antidiabetics, insulin, and non‐insulin injectable medications varies, as adherence to guidelines is difficult, given the potential changes in daily life after diagnosis [7, 8]. The Primary Health Care (PHC) work process should provide comprehensive diabetes care with a multidisciplinary approach focused on the unique needs of individuals, families, and communities [9]. In Brazil, diabetes programs often focus on refilling prescriptions. Policies that guarantee free access to diabetes medications are essential. At this point, it is crucial to focus on people's relationship with medications and the potential contributions of healthcare teams to streamline this process [10].

In this context, this study aimed to analyze adherence to insulin treatment in people with type 1 diabetes (T1D) or type 2 diabetes (T2D) in PHC in a Brazilian municipality, also aiming to investigate potential problems related to non‐adherence and generate hypotheses that will contribute to the planning of health actions to promote comprehensive diabetes care.

## 2 Methods

The data analyzed in this study are nested in the “Diabetes in Jardinópolis: Health survey of people with diabetes using insulin in primary health care in a municipality in São Paulo,” a cross‐sectional study based on a probability sample of the population of Jardinópolis, São Paulo state, Brazil. Data were collected in March 2023.

The study included men and women, aged 18 or older, with a medical diagnosis T1D or T2D in use of insulin and registered with primary care services in the municipality under study. The study excluded pregnant women, people who had discontinued insulin use on medical advice in the 7 days before the study, bedridden or homebound individuals, individuals with an acute infectious process, and those with a history of surgery or hospitalization within the 90 days before the study.

In calculating the sample size, the reference population of 834 people with T1D or T2D using insulin was considered, along with a frequency of 14.1% of glycemic control among people using insulin, a tolerable absolute error of 5% and a 95% confidence interval, resulting in 152 participants. Participants were obtained through probabilistic sampling [11].

Blood and urine samples were collected, as well as an ophthalmological examination of the fundus using a portable retinal camera on a smartphone. Face‐to‐face interviews were conducted at a health unit selected for the study, with the application of questionnaires whose data were collected and stored in REDCap at redcap.fmrp.usp.br. This platform is a secure web application for creating and managing online surveys and databases [12].

In the analysis of insulin therapy adherence, a score generated from questions in the “Insulin Treatment Adherence Measure (TAM),” a version validated in Brazil, was adopted. This scale consists of 7 items with a response pattern ranging from “always” to “never,” with scores ranging from 1 to 6, respectively. The overall mean of the instrument estimates adherence. The calculated score categorizes adherence to insulin therapy when the mean obtained is between 5 and 6 points and non‐adherence when the mean is less than 5 points [13].

The independent variables explored were gender (male and female), age group (under 45; 45–59; 60–74; and 75 and over), self‐reported ethnicity/skin color (White and Nonwhite), schooling (0; 1–9; 10–12; and 13 study years or more), economic class (A, B, C, and D/E) [14], self‐rated health (excellent, good, fair, poor, or very poor), alcohol abuse (yes or no) [15], tobacco use, self‐reported type of diabetes (1 or 2), disease length (1–10; 11–20; over 20 years, and does not know), self‐reported health conditions associated with diabetes (systemic arterial hypertension, dyslipidemia, overweight/obesity, kidney disease, ulcer/wound, and amputation), private health plan (yes or no), glycemic control with glycated hemoglobin less than 7% (yes or no) [16] and albuminuria (normal [< 30 mg/g]; microalbuminuria [30–299 mg/g] or macroalbuminuria [> 300 mg/g]) [17], diabetic retinopathy (yes or no) [18], number of insulin(s) used (1 or 2), use of NPH insulin (yes or no), use of regular insulin (yes or no), use of insulin and oral antidiabetic (yes or no).

Statistical analyses were performed using R software version 4.3.2 [19]. The readxl package was used for data import. Database management and organization were performed using the dplyr and tidyverse packages. Statistical procedures and logistic regression models were executed with the stats package, while the organization and presentation of results were performed using the broom and epiDisplay packages.

The normality of the variables was assessed through graphical inspection (Q–Q plot) and analysis of the skewness and kurtosis coefficients. The distributions showed an approximately symmetrical pattern, with no evidence of significant deviations from normality. Furthermore, considering the robust sample size (*n* = 152), the Central Limit Theorem guarantees that the distribution of the sample means satisfactorily approximates normality, ensuring the appropriateness of using parametric tests.

We calculated absolute and relative frequencies of variables with their respective 95% confidence intervals (95% CI). Poisson regression was used to estimate the prevalence ratio (PR) [20] of insulin therapy adherence among the variables of interest, both crude and adjusted for gender, age, and diabetes type. Pearson's chi‐square test was also employed to analyze the statistical significance of differences between categories. The median, interquartile range (IQR) mean, and respective standard deviation (SD) were calculated for insulin TAM‐related items. Student's *t*‐test was used to compare item means, using the item “Have you ever stopped taking insulin for diabetes, on your own initiative, because you felt worse?” as a reference. The level of statistical significance adopted was 5%.

The Research Ethics Committee of the “Dr. Joel Domingos Machado” School Health Center of the Ribeirão Preto School of Medicine of the University of São Paulo approved the research. The Certificate of Presentation of Ethical Appreciation N°64301322.0.0000.5414 and the Opinion N°5.748.306 were obtained. All participants signed the informed consent form.

## 3 Results

A total of 152 people with T1D or T2D using insulin participated in this study. A higher frequency of women was observed, with low income and education, without private health plan, self‐reported medical diagnosis of T2D, disease length of 21 years or more, inadequate glycemic control, no diabetic retinopathy, and normal albuminuria. Furthermore, most patients used one type of insulin, as well as oral antidiabetic medication combined with insulin (Table 1).

**TABLE 1:** Sample characterization and prevalence of adherence to insulin therapy according to sociodemographic, economic, clinical variables, and use of health services. Diabetes Jardinópolis Survey, São Paulo, Brazil, 2023. (*n* = 152).

Variables	Sample *n* (%)	Prevalence of adherence to insulin therapy (95% CI)^a^
Gender		
Female	96 (63.2)	47.9 (37.9–57.9)
Male	56 (36.8)	46.4 (33.3–59.4)
Age (years)		
< 45	18 (11.8)	38.8 (16.3–61.4)
45–59	57 (37.5)	49.1 (36.1–62.1)
60–74	60 (39.5)	46.6 (34.0–59.2)
> 75	17 (11.2)	52.9 (29.2–76.6)
Self‐reported ethnicity/skin color		
Nonwhite	93 (61.2)	41.9 (31.9–51.9)
White	59 (38.8)	55.9 (43.2–68.6)
Schooling (study years)		
0	21 (13.8)	66.7 (46.5–86.8)
1–9	96 (63.2)	47.9 (37.9–57.9)
10–12	28 (18.4)	32.2 (14.8–49.4)
≥ 13	7 (4.6)	42.8 (6.2–79.5)
Economic class [14]		
A	2 (1.3)	100.0 (34.2–100.0)
B	13 (8.6)	53.8 (26.7–80.9)
C	103 (67.8)	45.6 (36.1–55.2)
D/E	34 (22.3)	47.1 (30.2–63.8)
Self‐rated health		
Excellent	14 (9.2)	50.0 (23.8–76.2)
Good	61 (40.2)	44.2 (31.7–56.7)
Fair	48 (31.6)	52.1 (37.9–66.2)
Poor	15 (9.8)	60.0 (35.2–84.7)
Very poor	14 (9.2)	28.5 (4.9–52.2)
Alcohol abuse [15]		
No	119 (78.3)	49.5 (40.6–58.5)
Yes	33 (21.7)	39.4 (22.7–56.1)
Tobacco use		
No	133 (87.5)	48.8 (40.3–57.3)
Yes	19 (12.5)	36.8 (15.1–58.5)
Self‐reported type of diabetes		
1	10 (6.7)	50.0 (19.0–80.9)
2	142 (93.3)	47.2 (38.9–55.4)
Disease length (years)		
1–10	11 (7.24)	18.2 (0–4.9)
11–20	58 (38.16)	44.8 (32.0–57.6)
≥ 21	83 (54.60)	49.4 (38.6–60.1)
Self‐reported health conditions associated with diabetes		
Systemic arterial hypertension	108 (71.1)	52.7 (43.2–62.1)
Dyslipidemia	88 (57.9)	51.1 (40.6–61.5)
Overweight/obesity	66 (43.4)	45.4 (33.4–57.4)
Kidney disease	17 (11.2)	52.9 (29.2–76.6)
Ulcer/wound	18 (11.9)	27.7 (7.1–48.4)
Amputation	7 (4.6)	14.3 (2.56–51.3)
Private health plan		
No	128 (84.2)	53.9 (45.2–62.5)
Yes	24 (15.8)	12.5 (4.34–31.0)
Glycemic control [16]		
No	117 (76.9)	44.4 (35.4–53.4)
Yes	35 (23.1)	57.1 (40.7–73.5)
Albuminuria [17]		
Normal	86 (56.7)	54.6 (44.1–65.1)
Microalbuminuria	46 (30.2)	30.4 (17.1–43.7)
Macroalbuminuria	20 (13.1)	55.0 (33.1–76.8)
Diabetic retinopathy [18]		
No	109 (71.7)	50.4 (41.0–59.8)
Yes	43 (28.3)	39.5 (24.9–54.1)
Number of insulin(s) used		
1	80 (52.6)	51.2 (40.2–62.2)
2	72 (47.4)	43.0 (31.6–54.4)
NPH insulin use		
No	3 (1.9)	33.3 (0–86.6)
Yes	149 (98.1)	47.6 (39.6–55.6)
Regular use of insulin		
No	83 (54.6)	50.6 (39.8–61.3)
Yes	69 (45.4)	43.4 (31.7–55.17)
Use of insulin and oral antidiabetic drugs		
No	28 (18.4)	35.7 (17.9–53.4)
Yes	124 (81.5)	50.0 (41.1–58.8)

^a^ 95% confidence interval.

The prevalence of adherence to insulin therapy was estimated at 47.4% (95% CI 39.4–55.3). In the *t*‐test comparing the means of the items on the MAT insulin scale, with the reference question “Have you ever stopped taking insulin for your diabetes, on your own initiative, because you felt worse?”, an association was obtained with the items related to forgetfulness, carelessness with the time of insulin administration, failure to administer insulin when feeling better, and discontinuing treatment because you ran out of insulin (Table 2). An association was observed between adherence to insulin therapy and male, self‐reported Nonwhite, and self‐reported diagnosis of systemic arterial hypertension (Table 3).

**TABLE 2:** Measures of central tendency and *t*‐test among items on the Insulin Treatment Adherence Measure scale [13]. Diabetes Jardinópolis Survey, São Paulo, Brazil, 2023.

Insulin treatment adherence measurement scale items	Median (IQR)^a^	Mean (SD)^b^	*p* c
Have you ever stopped taking your diabetes insulin because you felt worse?	6 (3–6)	4.3 (1.9)	—
Have you ever forgotten to take your diabetes insulin?	4 (3–6)	4.0 (1.7)	0.03
Have you ever been careless with the timing of your diabetes insulin injections?	4 (2–6)	3.8 (1.8)	< 0.01
Have you ever stopped taking your diabetes insulin because you felt better?	4 (2–6)	4.0 (2.0)	0.04
Have you ever taken one or more units of diabetes insulin because you felt worse?	6 (3–6)	4.6 (1.8)	0.10
Have you ever stopped taking your diabetes treatment because you ran out of insulin?	6 (6–6)	5.4 (1.2)	< 0.01
Have you ever stopped taking your diabetes insulin for any reason other than your doctor's instructions?	4.5 (2–6)	4.2 (1.9)	0.32

^a^ Interquartile range.

^b^ Standard deviation.

^c^ Student's *t*‐test. Statistical significance is considered when *p* < 0.05.

**TABLE 3:** Association between adherence to insulin therapy and sociodemographic, economic, clinical variables, and use of health services. Diabetes Jardinópolis Survey, São Paulo, Brazil, 2023. (*n* = 152).

Variables	Crude PR^a^ (95% CI)^b^	*p* c	Adjusted PR^d^ (95% CI)	*p*
Gender		0.85		0.03
Female	1		1	
Male	0.94 (0.49–1.82)		6.35 (1.17–34.43)	
Age (years)		0.84		0.08
< 45	1		1	
45–59	1.52 (0.51–4.47)		1.18 (0.12–11.70)	
60–74	1.37 (0.47–4.03)		0.17 (0.01–2.00)	
> 75	1.77 (0.46–6.78)		0.72 (0–12.08)	
Self‐reported ethnicity/skin color		0.92		0.01
Nonwhite	0.57 (0.29–1.10)		1.62 (0.04–0.66)	
White	1		1	
Self‐rated health		0.44		0.61
Excellent	1		1	
Good	0.79 (0.25–2.54)		1.98 (0.27–14.45)	
Fair	1.09 (0.3–33.58)		1.64 (0.25–10.64)	
Poor	1.50 (0.34–6.53)		7.24(0.58–90.22)	
Very poor	0.40 (0.08–1.91)		1.83(0.11–29.66)	
Alcohol abuse [15]		0.29		0.03
No	1		1	
Yes	0.66 (0.30–1.45)		0.17 (0.03–0.83)	
Tobacco use		0.32		0.21
No	1		1	
Yes	0.61 (0.23–1.65)		0.34 (0.06–1.83)	
Self‐reported diabetes type		0.86		0.67
1	1		1	
2	0.89 (0.25–3.22)		0.50 (0.02–12.14)	
Disease length (years)		0.85		0.46
1–10	1		1	
11–20	0.98 (0.27–3.56)		4.21 (0.41–43.23)	
≥ 21	1.17 (0.33–4.14)		3.87 (0.35–42.22)	
Self‐reported systemic arterial hypertension		0.03		< 0.01
No	1		1	
Yes	2.16 (1.04–4.48)		1.34 (2.43–74.15)	
Self‐reported dyslipidemia		0.27		0.78
No	1		1	
Yes	1.43 (0.75–2.74)		1.17 (0.36–3.74)	
Self‐reported overweight/obesity		0.67		0.82
No	1		1	
Yes	0.87 (0.46–1.66)		1.15 (0.33–4.00)	
Self‐reported kidney disease		0.67		0.82
No	1		1	
Yes	1.29 (0.47–3.53)		0.69 (0.10–4.80)	
Self‐reported ulcer/wound		0.07		0.15
No	1		1	
Yes	0.38 (0.13–1.14)		0.25 (0.03–1.67)	
Self‐reported amputation		0.05		0.03
No	1		1	
Yes	0.17 (0.02–1.48)		0 (0–3.03)	
Private health plan		< 0.01		< 0.01
No	1		1	
Yes	0.12 (0.03–0.43)		0.03 (0.01–0.38)	
Glycemic control [16]		0.18		0.37
No	1		1	
Yes	1.67 (0.78–3.57)		1.88 (0.46–7.544)	
Albuminuria [17]		0.02		0.10
Normal	1		1	
Microalbuminuria	0.36 (0.17–0.77)		0.24 (0.09–0.96)	
Macroalbuminuria	1.01 (0.38–2.70)		0.80 (0.10–6.13)	
Diabetic retinopathy [18]		0.22		0.83
No	1		1	
Yes	0.64 (0.31–1.32)		0.80 (0.10–6.13)	
Number of insulin(s) used		0.46		0.73
1	1		1	
2	0.73 (0.31–1.71)		0.49 (0.007–30.79)	
NPH insulin use		0.61		0.76
No	1		1	
Yes	1.82 (0.16–20.51)		0.38 (0–203.41)	
Regular use of insulin		0.38		0.98
No	1		1	
Yes	0.75 (0.40–1.43)		1.05 (0.01–58.48)	
Use of insulin and oral antidiabetic drugs		0.168		0.02
No	1		1	
Yes	1.8 (0.77–4.21)		6.21 (0–0.99)	

^a^ Crude prevalence ratio.

^b^ 95% confidence interval.

^c^ Person's chi‐square. Statistical significance is considered when *p* < 0.05.

^d^ Adjusted prevalence ratio (gender, age, and self‐reported diabetes type).

## 4 Discussion

The prevalence of insulin therapy adherence was estimated at less than 50% in the PHC of the municipality under study, and this was a concern. Furthermore, the sociodemographic and clinical variables associated with adherence highlighted the need to address individual needs for comprehensive diabetes care.

A systematic review estimated the prevalence of adherence to insulin therapy among people with T1D or T2D at 55.37% (95% CI 48.55%–62.19%). The prevalence of adherence in low‐middle‐income countries was 56.79% (95% CI 27.85%–85.74%). The researchers highlighted that adherence to insulin therapy was low and emphasized the importance of health teams and health managers implementing strategic actions to promote adherence [21].

We observed a high frequency of participants who used the public health system exclusively for diabetes treatment. This fact highlights the relevance of strengthening the Unified Health System (SUS) and especially PHC, since the prevention and management of chronic noncommunicable conditions, in a well‐structured system, can curb hospitalization costs and diabetes‐related complications [22].

Adherence to insulin therapy was positively associated with males. Men tend to use health services less and are more vulnerable to expressing difficulties related to treatment. Therefore, this finding reveals different hypotheses, as adherence may have been overestimated in this group, which was also observed in another Brazilian study in PHC [23].

The association between alcohol abuse and low adherence was notable in this study, in agreement with other findings in the literature. A study conducted in Ribeirão Preto, São Paulo, estimated that 19.2% (95% CI 15.0%–23.4%) of older adults with T2D engaged in alcohol abuse. Furthermore, a negative association was observed between alcohol abuse and adherence to drug therapy (PR = 0.55; 95% CI 0.36–0.86) [24].

The frequency of alcohol abuse and non‐adherence to medication among those with a high consumption pattern deserves attention from PHC health teams, as it can lead to diabetes complications. Alcohol is known to interfere with glycemic control, resulting in both hypoglycemia and hyperglycemia. This lack of control potentially harms individuals, especially those on continuous insulin therapy [25].

Diabetes and its complications have been responsible for high mortality in Brazil in recent years [26]. Among the complications, diabetes mainly affects vision, kidneys, peripheral, coronary, and cerebral circulation, in addition to increasing the likelihood of developing ulcers and, consequently, suffering amputations. National data have shown that the most prevalent are diabetic neuropathy and retinopathy [10].

The relationship between amputation and non‐adherence to insulin therapy is an important issue in diabetes management, especially among people with T2D, most of whom are older adults. Amputation can be a severe complication of diabetes, often resulting from infections, foot ulcers, and circulatory problems related to inadequate glycemic control. People who do not adhere closely to their insulin therapy regimens may experience glycemic spikes, which increase the risk of micro‐ and macro‐vascular complications, including problems that can lead to amputation [10].

Complication prevention should occur through disease management in PHC with the commitment of the multidisciplinary team to screen for diabetic foot, stratify risk, treat ulcers and other complications, as well as guide daily foot care [9]. However, data from the 2013 and 2019 National Health Surveys showed that only 29.1% and 31%, respectively, of Brazilians participating in the study underwent foot examinations by health professionals in the 12 months before the interview [27].

Systemic arterial hypertension and insulin therapy adherence were positively associated in this study. There are differing views on this matter in the literature and healthcare practice [28]. Notably, people with multiple health conditions and taking different medications may have difficulty following their medication regimens. Therefore, the mobilization of healthcare teams, along with families and caregivers, is essential. On the other hand, two high‐impact chronic conditions can encourage adherence, since the lack of adequate drug therapy management can entail a more significant risk of poor clinical outcomes [29].

A negative association was found between participants with a private health plan and adherence. The hypothesis is that longitudinality, an attribute of PHC, may still be compromised in private health insurance services. The SUS knowingly provides people with chronic noncommunicable conditions with a care network that encompasses prevention, diagnosis, treatment, monitoring, and health education, with a focus on PHC. National data from 2019 estimated that approximately 66.5% of people with diabetes underwent SUS follow‐up in the months before the survey [30]. This fact highlights the importance of strengthening the public health system, and especially PHC, since the prevention and management of diabetes, in a well‐structured system, can result in reduced costs with hospitalizations, as well as complications [31].

Insulin therapy is one of the main strategies in the treatment of diabetes. It is vital in T1D to enable glycemic control and prevent complications associated with the disease [32]. However, adherence is often compromised by several variables. Insulin treatment is complex and requires individuals to understand the different types of insulin, administration schedules, injection sites and techniques, carbohydrate counting, and blood glucose monitoring [33]. The need to adjust insulin doses based on diet, exercise, comorbidities, and stress levels makes treatment management even more challenging. Therefore, the PHC team should necessarily manage drug therapy, identifying barriers to adherence and proposing care plans that encourage empowerment and self‐care [34].

Among the limitations, other studies on insulin therapy adherence used different measurement instruments. Most were conducted in specialized health services and often involved people with T1D or T2D. Therefore, comparisons were challenging. Another limitation was recall bias, as participants may not have reported the specific characteristics of drug therapy in their daily lives. However, this study used an instrument translated and validated in Portuguese that could analyzed adherence in a sample of people with T1D and T2D from PHC.

## 5 Conclusion

Adherence to insulin therapy was low, and this context can lead to acute and chronic complications with varying impacts, including increased healthcare costs. The identified barriers suggest a multidisciplinary approach in PHC for managing drug therapy to enable diabetes control. Furthermore, we propose to strengthen health education initiatives that encourage self‐care and empowerment among people using insulin.

## Author Contributions

**Lívia Maria Ferrante Vizzotto Consoli:** conceptualization, investigation, writing – original draft, methodology, validation, visualization, writing – review and editing, formal analysis, data curation, resources. **Rosiane Chiaroti:** conceptualization, investigation, writing – original draft, methodology, validation, visualization, writing – review and editing, formal analysis, data curation, resources. **Lorrana Luysse dos Anjos Assis:** conceptualization, investigation, writing – original draft, methodology, validation, visualization, writing – review and editing, formal analysis, data curation, resources. **Vanessa Patrícia Pereira Motozo:** conceptualization, investigation, writing – original draft, methodology, validation, visualization, writing – review and editing, formal analysis, data curation, resources. **Francisco Barbosa‐Junior:** conceptualization, investigation, writing – original draft, methodology, validation, visualization, writing – review and editing, formal analysis, software, data curation, resources. **Rinaldo Eduardo Machado de Oliveira:** conceptualization, investigation, funding acquisition, writing – original draft, writing – review and editing, visualization, validation, methodology, project administration, formal analysis, software, resources, supervision, data curation.

## Ethics Statement

The study was approved by the Research Ethics Committee of the Dr. Joel Domingos School Health Centre of the Ribeirão Preto Medical School of the University of São Paulo following the recommendations of the National Research Ethics Commission and according to protocol number 466/2012. Presentation Certificate of Ethics Appreciation under submission numbers 64301322.0.0000.5414 and 5748306 was obtained.

## Consent

All the participants of the study freely signed an informed consent form.

## Conflicts of Interest

The authors declare no conflicts of interest.

## Data Availability Statement

The data that support the findings of this study are available from the corresponding author upon reasonable request.

## Transparency Statement

The lead author Rinaldo Eduardo Machado de Oliveira affirms that this manuscript is an honest, accurate, and transparent account of the study being reported; that no important aspects of the study have been omitted; and that any discrepancies from the study as planned (and, if relevant, registered) have been explained.
